# Microbial Spectrum of Cholesteatomatous Otitis Media and Its Correlation With Cholesteatoma Staging: A Cross-Sectional Analytical Study

**DOI:** 10.7759/cureus.114256

**Published:** 2026-08-10

**Authors:** Sahil Chauhan, Shaila Sidam, Vikas Gupta, Arati A Bhadade, Ujjawal Khurana, Anjan K Sahoo, Aparna Chavan

**Affiliations:** 1 Otolaryngology - Head and Neck Surgery, All India Institute of Medical Sciences, Bhopal, Bhopal, IND; 2 Otolaryngology, All India Institute of Medical Sciences, Bhopal, Bhopal, IND; 3 Microbiology, All India Institute of Medical Sciences, Bhopal, Bhopal, IND; 4 Pathology and Laboratory Medicine, All India Institute of Medical Sciences, Bhopal, Bhopal, IND

**Keywords:** biofilm, cholesteatoma, chronic suppurative otitis media, eaono/jos staging, microbial profile, middle ear infection

## Abstract

Background: Cholesteatomatous chronic otitis media is a locally destructive condition in which persistent bacterial and fungal infection is thought to cause chronic inflammation and osteolytic activity, driving cholesteatoma progression; however, whether the microbial profile correlates with disease severity remains unclear.

Objective: This study aims to characterize the microbiological profile of cholesteatomatous otitis media and evaluate its relationship with cholesteatoma stage as defined by the European Academy of Otology and Neurotology/Japan Otological Society (EAONO/JOS) staging system.

Methods: In this hospital-based prospective cross-sectional analytical study conducted over 18 months at a tertiary care institute in Central India, 72 patients undergoing tympanomastoid surgery for chronic otitis media with cholesteatoma were enrolled. Cholesteatoma tissue obtained intraoperatively was submitted for aerobic culture, anaerobic culture, and fungal evaluation (KOH mount and culture). Disease stage was assigned using combined radiological and intraoperative findings per the EAONO/JOS (2017) system. Associations between microbiological findings and stage were assessed using Fisher's exact test and Spearman's rank correlation, with p<0.05 considered significant.

Results: Mean age was 25.36 ± 11.56 years (range 6-55), with a near-equal gender distribution: 35 males (48.6%) and 37 females (51.4%). Stage II disease was most common in 40 cases (55.6%), followed by Stage III in 26 cases (36.1%). Aerobic bacterial growth was identified in 61 patients (84.7%), fungal elements in 21 (29.2%), and anaerobic bacteria in 2 (2.8%). No statistically significant association was found between disease stage and aerobic (p=0.407), anaerobic (p=0.440), or fungal (p=0.349) growth. Spearman's correlation showed only weak, non-significant relationships between stage and aerobic bacteria (ρ=0.12, p=0.31), anaerobic bacteria (ρ=−0.10, p=0.42), and fungal positivity (ρ=−0.17, p=0.14).

Conclusion: The microbial profile in cholesteatomatous otitis media was not significantly associated with disease stage; rather, anatomical severity is driven more by the host inflammatory response, biofilm formation, and the intrinsic osteolytic behaviour of cholesteatoma than by the specific organisms isolated. Microbiological culture remains valuable for guiding antimicrobial therapy but should not be used as a marker of disease severity.

## Introduction

Chronic suppurative otitis media (CSOM) is an inflammatory disease of the middle ear cleft and mastoid characterized by persistent ear discharge through a tympanic membrane perforation for more than six weeks and is commonly associated with hearing loss [1,2]. It remains a major global health problem, particularly in developing countries, where it is an important cause of preventable hearing impairment and long-term morbidity [3]. Progressive disease may result in bone erosion, ossicular destruction, labyrinthine fistula, facial nerve palsy, and intracranial complications [4].

Cholesteatomatous otitis media is characterized by the presence of keratinizing squamous epithelium within the middle ear cleft and mastoid cavity [5]. The aggressive behaviour of cholesteatoma is incompletely understood; however, persistent bacterial infection and chronic inflammation contribute significantly to disease progression. This infection and inflammation can be due to toxins released from bacteria, inflammatory cytokines, and proteolytic enzymes released from either microorganisms or the host itself, leading to osteolytic activity and bone resorption [4,5].

In cholesteatomatous otitis media, the most frequently detected aerobic microorganisms are *Pseudomonas aeruginosa*, *Staphylococcus aureus*, and *Proteus mirabilis* [6-8]. Other reported anaerobic bacteria are *Peptostreptococcus* and *Bacteroides* species, together with fungi including *Candida albicans*, *Candida parapsilosis*, and *Aspergillus niger*, which have also been identified [9,10]. These microorganisms have the ability to form biofilms, which are structured, enclosed communities within an extracellular polymeric matrix that helps them enhance resistance to host immune defences and antimicrobial agents. The biofilm formation within poorly ventilated air cells such as the sinus tympani and mastoid air cells contributes to persistence of infection, recurrence, and poor response to treatment [10,11].

The extent and severity of cholesteatoma are assessed using the European Academy of Otology and Neurotology/Japan Otological Society (EAONO/JOS) staging system, which categorizes disease according to involvement of four anatomical subsites: the tympanic cavity (T), attic (A), mastoid (M), and supratubal recess or sinus tympani (S) [12]. This standardized classification facilitates disease evaluation, prognostication, comparison of clinical outcomes, and reporting across studies.

As microbial infection contributes to the pathogenesis and progression of cholesteatoma, clarifying the relationship between microbial profiles and cholesteatoma stage may provide insights into disease behaviour, guide antimicrobial therapy, and assist in predicting disease severity.

Therefore, the present study aimed to investigate the microbiological profile of cholesteatomatous otitis media and evaluate its relationship with cholesteatoma staging according to the EAONO/JOS classification system.

## Materials and methods

This hospital-based prospective cross-sectional analytical study was conducted at the All India Institute of Medical Sciences, Bhopal, a tertiary care institute in Central India, over a period of 18 months (23 August 2023 to 24 February 2025). Ethical approval was obtained from the Institutional Ethics Committee (Reference No. AIIMS/BPL/IHECSR/JAN/23/PG/19).

A consecutive sampling technique was employed for patient enrolment. Over the 18-month study period, an initial cohort of 100 patients diagnosed with chronic otitis media (COM) squamosal disease, presenting for evaluation and scheduled to undergo tympanomastoid surgery, were screened for eligibility. Of these, 28 patients were excluded based on the predefined exclusion criteria. The excluded participants comprised three patients with a history of head injury following road traffic accidents, 20 patients with various systemic comorbidities, three patients with a history of previous mastoid surgery, and two patients using topical ear drops. These exclusions were undertaken to ensure that the final cohort was free of confounding factors that could impact the assessment of the microbiological profile and its relationship with cholesteatoma staging. Following these exclusions, sample size was determined by feasibility over the 18-month recruitment period. A final sample of 72 patients diagnosed with COM squamosal disease, who either had visible cholesteatoma or were suspected of harbouring cholesteatoma and subsequently underwent tympanomastoid surgery, were enrolled and constituted the study population.

Patients were excluded if they had received systemic or topical antibiotics or steroids within 15 days prior to surgery, had known systemic inflammatory or autoimmune disorders, refused consent, had undergone previous ear surgery, or were deemed unfit for surgery.

The assessment and extent of cholesteatoma was done by using the EAONO/JOS 2017 staging system [12]. This classification categorizes cholesteatoma into four stages based on involvement of the attic (A), tympanic cavity (T), mastoid (M), and the presence of complications (C). Stage I represents disease confined to the primary site of origin, whereas Stage II indicates extension to two or more anatomical sites. Stage III includes cases associated with extracranial complications such as facial nerve palsy, labyrinthine fistula, or postauricular abscess, while Stage IV comprises cases with intracranial complications, including meningitis, brain abscess, or lateral sinus thrombosis. Final staging was assigned by the operative surgeon, combining preoperative and radiological findings with intraoperative surgical findings, who was blinded to microbiological culture and results in order to avoid bias. In case of discordance, intraoperative findings were considered definitive.

A detailed history and otological examination was done of all participants. Patients suspected of COM squamosal disease underwent high-resolution computed tomography (HRCT) of the temporal bone, slice thickness (0.6-1 mm), and imaging planes (axial+coronal reformats) to look for the extent of cholesteatoma, status of ossicular chain, any bony dehiscence or erosion, and intracranial or extracranial complications. Scans were reported by a single radiologist who was blinded to intraoperative or microbiological findings.

All patients scheduled for surgical intervention underwent preoperative evaluation on an outpatient basis, including routine hematological investigations in the form of a complete blood count (CBC) and pure-tone audiometry. Tympanomastoid surgery was performed as per the planned surgical approach based on preoperative radiological findings and intraoperative disease extent. Intraoperative findings were noted, including cholesteatoma extension, ossicular chain status, involvement of the middle ear cleft, and presence of complications.

Intraoperatively, cholesteatoma tissue was aseptically harvested and divided into three separate sterile containers for microbiological analyses. The aerobic microbiological specimen was transported in sterile normal saline for Gram staining, aerobic culture, and antimicrobial susceptibility testing. For fungal evaluation, the specimen was transported in sterile normal saline for potassium hydroxide (KOH) mount and fungal culture. The anaerobic microbiological specimen was transported in Robertson’s cooked meat medium for anaerobic culture and sensitivity testing. For aerobic culture, the specimens were inoculated onto blood agar and MacConkey agar and incubated at 35-37°C for 24-48 hours. Fungal culture specimens were inoculated onto Sabouraud dextrose agar and incubated at 25-28°C and/or 37°C for up to 2-4 weeks. The anaerobic specimen received in Robertson's cooked meat medium was subcultured onto selective and non-selective media containing a metronidazole (5 µg) disc and then incubated in an anaerobic jar at 37°C for 48-72 hours. All isolates were identified by the MALDI-TOF MS method. The distinction between pathogen and contaminant was made using the following criteria: an organism was considered a true pathogen if the isolated organism was grown in pure culture and was concordant with Gram stain findings. Growth of typical skin/environmental flora in scant amounts without corroborating Gram stain findings was considered a contaminant.

The staging of cholesteatoma was done using combined radiological and intraoperative findings; subsequently, the relationship between microbial infection and cholesteatoma stage was evaluated.

Data were entered into Microsoft Excel (Microsoft Corporation, Redmond, Washington) and analyzed using IBM SPSS Statistics for Windows, Version 29 (Released 2023; IBM Corp., Armonk, New York). Categorical variables were summarized as frequencies and percentages, whereas continuous variables were expressed as mean ± standard deviation (SD). Associations between cholesteatoma staging (EAONO/JOS) with microbiological findings were analyzed using Fisher’s exact test and correlation with Spearman's rank correlation. A p-value of less than 0.05 was considered statistically significant.

## Results

The demographic profile of the study participants is summarized in Table 1. The age of the participants ranged from 6 to 55 years, with a mean age of 25.36 ± 11.56 years. The majority of patients belonged to the 18-45 years age group (52/72, 72.2%), followed by those aged less than 18 years (16/72, 22.2%). Only four patients (5.6%) were older than 45 years. The study population showed a nearly equal gender distribution, comprising 37 females (51.4%) and 35 males (48.6%), with no marked gender predominance observed.

**Table 1:** Demographic Characteristics of the Study Participants (n = 72)

Characteristic	Value
Age Group (years)	
<18	16 (22.2%)
18–45	52 (72.2%)
>45	4 (5.6%)
Age (years)	25.36 ± 11.56 (6–55)
Gender	
Male	35 (48.6%)
Female	37 (51.4%)

The distribution of cholesteatoma stages according to gender is presented in Table 2. Stage II was the most common clinical stage, accounting for 40 patients (55.6%), followed by Stage III with 26 patients (36.1%). Stage I and Stage IV disease were less frequent, comprising four (5.6%) and two (2.8%) patients, respectively. Among patients with Stage II disease, females constituted a slightly higher proportion than males (57.5% vs. 42.5%). In contrast, Stage III disease was more prevalent among males, who accounted for 61.5% of cases in this stage. Stage I disease showed an equal distribution between males and females. Notably, both patients with Stage IV disease were female.

**Table 2:** Distribution of Clinical Stages of Cholesteatoma According to Gender (n = 72)

Clinical Stage	Male, n (%)	Female, n (%)	Total, n (%)
I	2 (50.0)	2 (50.0)	4 (5.6)
II	17 (42.5)	23 (57.5)	40 (55.6)
III	16 (61.5)	10 (38.5)	26 (36.1)
IV	0 (0.0)	2 (100.0)	2 (2.8)
Total	35 (48.6)	37 (51.4)	72 (100.0)

Table 3 compares microbiological findings (aerobic, anaerobic, and fungal growth) across disease stages I-IV.

**Table 3:** Association Between Microbiological Profile and Clinical Stage of Cholesteatoma (n = 72) Statistical test: Fisher's exact test was used for comparison. Significance level: p < 0.05.

Microbiological Profile	Status	Stage I (n=4)	Stage II (n=40)	Stage III (n=26)	Stage IV (n=2)	Total	Fisher’s Exact Test (p-value)
Aerobic growth	Present	4	31	24	2	61	0.407
	Absent	0	9	2	0	11	
Anaerobic growth	Present	0	2	0	0	2	0.440
	Absent	4	38	26	2	70	
Fungal positivity	Positive	2	13	6	0	21	0.349
	Negative	2	27	20	2	51	

All three p-values (aerobic: 0.407, anaerobic: 0.440, fungal: 0.349) are greater than 0.05, indicating that there is no statistically significant association between disease stage and any of the microbiological profiles in this cohort.

A weak positive correlation was observed between aerobic bacterial growth and disease stage (ρ = 0.12, p = 0.31), while weak negative correlations were observed for anaerobic bacteria (ρ = -0.10, p = 0.42) and fungal positivity (ρ = -0.17, p = 0.14) (Table 4). None of the associations demonstrated statistical significance, indicating that clinical stage was not meaningfully associated with aerobic, anaerobic, or fungal microbiological profiles in this cohort. The findings suggest that microbial presence is likely independent of disease stage rather than progressively influenced by disease severity.

**Table 4:** Spearman's Rank Correlation Between Clinical Stage and Microbiological Profile in Cholesteatoma (n = 72) Values represent Spearman’s rank correlation coefficient (ρ). Clinical stage was treated as an ordinal variable (Stage I–IV). Microbiological variables were coded as binary (0 = absent/negative, 1 = present/positive). Two-tailed significance tested; df = 70. Statistical significance set at p < 0.05.

Variables	Spearman’s ρ	p-value
Aerobic bacteria	0.12	0.31
Anaerobic bacteria	-0.10	0.42
Fungal positivity	-0.17	0.14

## Discussion

Cholesteatomatous otitis media is a chronic middle ear disease characterized by chronic infection, inflammation, and the ability to form a cholesteatoma, which can cause bone erosion leading to serious complications. Microbial infection may affect the pathogenesis, chronicity, and progression of the disease, influencing the clinical stage and treatment outcomes. Identification of the microbial profile associated with cholesteatomatous otitis media and its relationship to cholesteatoma staging may improve understanding of disease progression, facilitate appropriate antimicrobial therapy, and assist in predicting disease severity. Therefore, the present cross-sectional analytical study was undertaken to evaluate the microbial spectrum in cholesteatomatous otitis media and to examine its association with cholesteatoma staging.

In the present study, the mean age of patients with cholesteatomatous otitis media was 25.36 ± 11.56 years, and the majority of cases occurred in the 18-45-year age group (Table 1). This finding is consistent with previous studies reporting that acquired cholesteatoma predominantly affects adolescents and young adults. Recent epidemiological studies have similarly reported peak occurrence of cholesteatoma during the second and third decades of life, with mean ages ranging from approximately 21 to 31 years [13,14].

Gender distribution in our cohort was nearly equal, with females accounting for 51.4% and males 48.6% of cases (Table 2). Similar observations have been reported in studies demonstrating no significant gender predilection among patients with cholesteatoma [13,14]. While some epidemiological studies have reported a slight male predominance, the differences are generally modest and are unlikely to be of major clinical significance [13].

In the present study, Stage II cholesteatoma was the most common presentation, accounting for more than half of all cases, followed by Stage III disease. This finding suggests delayed presentation in developing countries, where patients seek medical attention after the disease has progressed beyond the earliest stage, due to delayed healthcare access, awareness, and prolonged duration of symptoms, often resulting in presentation at intermediate or advanced stages of cholesteatoma [13,14].

In the present study, the association between microbiological profile and clinical staging of cholesteatomatous otitis media (Table 3) showed that aerobic bacterial growth was the most frequently isolated microbiological finding (61, 84.7%), followed by fungal positivity (21, 29.2%), while anaerobic growth was rare (2, 2.8%). However, statistical analysis using Fisher’s exact test showed no significant association between microbiological profile and clinical stage (aerobic growth p = 0.407, anaerobic growth p = 0.440, fungal positivity p = 0.349). These findings suggest that disease severity in cholesteatomatous otitis media may not be directly dependent on the type of microbial flora present, but rather on complex interactions between host immunity, chronic inflammation, and anatomical disease progression [13,15].

The predominance of aerobic organisms in our cohort is consistent with current literature on CSOM microbiology, where aerobic gram-negative bacteria such as *Pseudomonas aeruginosa* and *Proteus mirabilis* are the most commonly isolated pathogens [8,16]. Their presence across all disease stages supports the finding that chronic middle ear infections are predominantly due to biofilm-forming aerobic bacteria, which are capable of surviving in hostile environments of the middle ear cleft [13,16]. This leads to chronicity and treatment resistance in CSOM, particularly in cholesteatoma cases. Fungal growth was observed in 21 (29.2%) of the cases, with a higher occurrence in Stage II and Stage III disease. This finding aligns with the view of fungal co-infection in chronic ear cases with prolonged duration, prior antibiotic usage, and exposure to humid environments [15,16]. However, we found that fungal positivity did not show a significant correlation with disease stage, suggesting that fungal colonization may be secondary rather than a primary cause of disease progression in cholesteatomatous otitis media.

Anaerobic organisms were rarely isolated (2, 2.8%), which aligns with contemporary studies reporting low detection rates in routine cultures due to their fastidious nature and requirement for specialized transport and culture techniques [16]. Despite this, we found no significant association between anaerobic growth and clinical staging.

The lack of statistically significant association between microbiological profile and clinical stage suggests that disease severity is more likely affected by host-related factors such as impaired mucociliary clearance due to eustachian tube dysfunction, inflammation, and osteolytic activity due to cholesteatoma itself [13,15,17]. Recent evidence also supports the local aggressiveness of cholesteatoma, characterized by keratin deposition and bone destruction rather than being purely driven by infection [13,15].

Clinically, these findings suggest that antimicrobial therapy should be based on a local antibiogram rather than the stage of disease. Hence, surgical management remains the definitive treatment for cholesteatoma, as antibiotics play an adjunctive role in controlling infection due to biofilms and chronic inflammation [13,17]. This emphasizes that an integrated medical and surgical approach is required to control disease progression.

The present study also assessed the relationship between the clinical stage of cholesteatomatous otitis media and the microbiological profile using Spearman’s rank correlation analysis. The results demonstrated a very weak positive correlation between clinical stage and aerobic bacterial growth (r = 0.12) and weak negative correlations with anaerobic bacteria (r = −0.10) and fungal positivity (r = −0.17). Overall, these correlations were minimal, suggesting that progression of clinical stage is not strongly associated with the type of microorganisms isolated from the ear discharge (Table 4). These findings suggest that disease severity in cholesteatoma is mostly driven by chronic pathological processes rather than specific microbial patterns.

The weak positive correlation between clinical stage and aerobic bacterial growth reflects the presence of aerobic organisms across all stages of disease. Aerobic bacteria, *Pseudomonas aeruginosa* and *Staphylococcus aureus*, are well-documented as dominant pathogens in cholesteatomatous otitis media [8,16]. However, their near-universal presence across stages reduces their discriminatory value in predicting disease severity. Recent studies have similarly reported that while aerobic bacteria contribute to persistent infection, they do not significantly influence the anatomical extent of disease progression [13,16].

The weak negative correlation observed between clinical stage and anaerobic bacterial growth may be due to the overall low prevalence of anaerobes in the present study. This may be due to their detection remaining limited due to culture constraints. Molecular studies have suggested that anaerobes may contribute to chronic inflammation; however, their role in determining clinical severity or cholesteatoma staging remains unclear [15,16].

Similarly, the weak negative correlation between fungal positivity and clinical stage suggests that it is unlikely to play a direct role in disease progression. In prolonged antibiotic usage, humid environments, and with impaired immunity, *Aspergillus* and *Candida* are often secondary colonizers [15,16]. Their presence across various stages, without a clear trend towards advanced disease, further supports their opportunistic nature rather than a causative role in disease progression.

These findings suggest that microbial infection alone does not correlate with the staging of cholesteatomatous otitis media. This supports the view that cholesteatoma is a locally destructive disease due to keratinizing squamous epithelium, inflammation, and enzymatic bone resorption rather than a purely infection-driven process [13,15,17]. Hence, we speculate that the more critical determinants of disease persistence and progression may be biofilm formation and altered host immune response rather than microbial infection [13,15,16].

Clinically, we can conclude that microbial culture results should guide antimicrobial therapy for infection control rather than be used as an indicator for disease severity. The weak correlations observed reinforce the need for early surgical and disease management rather than relying on microbiological findings to stage the disease [13,17].

The present study has limitations. For example, the overall sample size was adequate for the primary analysis, but the distribution of cases across cholesteatoma stages was uneven, and several stage-wise subgroups contained relatively few patients. This limited statistical power for detecting associations within individual subgroups. It was conducted at a single tertiary care centre with a relatively modest sample size, which may limit the generalizability of the findings. The cross-sectional study design permits assessment of the association between the microbial spectrum and cholesteatoma staging but does not establish a causal relationship. Although microbiological culture techniques and standardized clinical and radiological criteria were used for organism identification and disease staging, the possibility of sampling errors, culture-negative infections, and observer variability cannot be completely excluded. Furthermore, prior or undocumented antibiotic use may have influenced the microbiological yield, potentially affecting the observed microbial spectrum. These limitations, particularly the constraints on subgroup power, should be addressed in future work through larger, multicentre, prospective, and blinded studies incorporating molecular or advanced microbiological techniques to validate and extend the present findings.

## Conclusions

In the present study, the most predominantly seen were aerobic organisms, followed by fungal organisms, and then infrequently seen were anaerobic bacteria. Although aerobic bacteria were seen across all stages, we found no statistically significant correlation between the microbiological spectrum and cholesteatoma staging. Similarly, correlation analysis found only a weak relationship between microbial profile and disease stage, suggesting that host factors, inflammation, biofilm formation, and pathological behaviour of the disease affect the anatomical extent and severity of cholesteatoma rather than the organism isolated. These interpretations suggest that microbiological culture is helpful in guiding antimicrobial therapy rather than in assessing disease severity or predicting the stage of cholesteatoma. Early diagnosis, timely surgical intervention, and appropriate antimicrobial management based on culture sensitivity remain essential for achieving optimal clinical outcomes. Further large-scale, multicentre prospective studies incorporating molecular microbiological techniques are warranted to better elucidate the role of microbial communities in the pathogenesis and progression of cholesteatomatous otitis media.

## References

[1] Bhutta MF, Leach AJ, Brennan-Jones CG (2024). Chronic suppurative otitis media. Lancet.

[2] Mittal R, Lisi CV, Gerring R (2015). Current concepts in the pathogenesis and treatment of chronic suppurative otitis media. J Med Microbiol.

[3] Monasta L, Ronfani L, Marchetti F (2012). Burden of disease caused by otitis media: systematic review and global estimates. PLoS One.

[4] Lei Y, An J, Ren Q, Wang M, Gao M (2023). Expression of MMP-14 and its role in bone destruction in middle ear cholesteatoma: a prospective observational study. Medicine (Baltimore).

[5] Kuo CL, Shiao AS, Yung M (2015). Updates and knowledge gaps in cholesteatoma research. Biomed Res Int.

[6] Alam M, Beig S, Sultan A, Chandra K (2022). Bacteriological and antibiotic sensitivity profile of cholesteatomatous otitis media in pediatric and adult population: prevailing scenario in Northern India. Indian J Otolaryngol Head Neck Surg.

[7] Sammal M, Pant B, Negi N, Sikarwar V (2024). Current trends in clinico-bacteriological profile and antimicrobial susceptibility pattern in active chronic suppurative otitis media (safe and unsafe) at a tertiary care center in Uttarakhand: an observational study. Cureus.

[8] Alam M, Sultan A, Chandra K (2022). Microbiological assessment of chronic otitis media: aerobic culture isolates and their antimicrobial susceptibility patterns. Indian J Otolaryngol Head Neck Surg.

[9] Neeff M, Broderick D, Douglas RG, Biswas K (2024). Anaerobic bacteria dominate the cholesteatoma tissue of chronic suppurative otitis media patients. Microb Pathog.

[10] Mujahid ZA, Palal SS, Gopan G, Ramabhadraiah AK (2024). Biofilm producing organisms and their antibiotic sensitivity in chronic suppurative otitis media: a cross-sectional study. Indian J Otolaryngol Head Neck Surg.

[11] Saunders J, Murray M, Alleman A (2011). Biofilms in chronic suppurative otitis media and cholesteatoma: scanning electron microscopy findings. Am J Otolaryngol.

[12] Yung M, Tono T, Olszewska E (2017). EAONO/JOS joint consensus statements on the definitions, classification and staging of middle ear cholesteatoma. J Int Adv Otol.

[13] Patel TR, Welch CM (2025). The science of cholesteatoma. Otolaryngol Clin North Am.

[14] Orobello N, Harrington C, Reilly BK (2022). Updates in paediatric cholesteatoma. Curr Opin Otolaryngol Head Neck Surg.

[15] Fujikawa T, Tanimoto K, Kawashima Y (2022). Cholesteatoma has an altered microbiota with a higher abundance of Staphylococcus species. Laryngoscope Investig Otolaryngol.

[16] Khairkar M, Deshmukh P, Maity H, Deotale V (2023). Chronic suppurative otitis media: a comprehensive review of epidemiology, pathogenesis, microbiology, and complications. Cureus.

[17] Pandiangan M, Gaffar M, Djamin R, Zainuddin AA, Sjahril R, Akil MA (2026). Exploring the link between bacterial biofilm and disease severity in cholesteatoma-associated CSOM. Medeni Med J.

